# Taekwondo Training Improves Balance in Volunteers Over 40

**DOI:** 10.3389/fnagi.2013.00010

**Published:** 2013-03-13

**Authors:** G. Pons van Dijk, A. F. Lenssen, P. Leffers, H. Kingma, J. Lodder

**Affiliations:** ^1^Department of Neurology, School for Mental Health and Neuroscience, University Hospital MaastrichtMaastricht, Netherlands; ^2^Department of Physical Therapy, Maastricht University Medical CenterMaastricht, Netherlands; ^3^Department of Epidemiology, Faculty of Health, Medicine and Life Sciences, School for Public Health and Primary Care (CAPHRI), Maastricht University Medical CentreMaastricht, Netherlands; ^4^Department of Clinical Vestibulogy, ENT-Department, Maastricht University Medical CentreMaastricht, Netherlands

**Keywords:** hard martial arts, Taekwondo, senior citizens, static balance, dynamic balance

## Abstract

Balance deteriorates with age, and may eventually lead to falling accidents which may threaten independent living. As Taekwondo contains various highly dynamic movement patterns, Taekwondo practice may sustain or improve balance. Therefore, in 24 middle-aged healthy volunteers (40–71 year) we investigated effects of age-adapted Taekwondo training of 1 h a week during 1 year on various balance parameters, such as: motor orientation ability (primary outcome measure), postural and static balance test, single leg stance, one leg hop test, and a questionnaire. *Motor orientation ability* significantly increased in favor of the antero-posterior direction with a difference of 0.62° toward anterior compared to pre-training measurement, when participants corrected the tilted platform rather toward the posterior direction; female gender being an independent outcome predictor. On *postural balance measurements* sway path improved in all 19 participants, with a median of 9.3 mm/s (range 0.71–45.86), and sway area in 15 participants with 4.2 mm^2^/s (range 17.39–1.22). *Static balance* improved with an average of 5.34 s for the right leg, and with almost 4 s for the left. Median *single leg stance duration* increased in 17 participants with 5 s (range 1–16), and in 13 participants with 8 s (range 1–18). The average *one leg hop test distance* increased (not statistically significant) with 9.5 cm. *The questionnaire* reported a better “ability to maintain balance” in 16. In conclusion, our data suggest that age-adapted Taekwondo training improves various aspects of balance control in healthy people over the age of 40.

## Introduction

Maintenance of balance is a complex process, and depends on proper integration of various functional systems, such as the somatosensory, visual, vestibular, and cerebellar system. Furthermore, it requires functional integrity of the peripheral neuromuscular system (Nasher, 1997). These various components deteriorate with age, and may eventually lead to falling accidents and threaten independent living. Vestibular function, for example, is severely reduced in over a third of people older than 65 (Ishiyama, 2009). Consequences of balance deterioration are an important cause of morbidity among the elderly (Wolter and Studenski, 1996). However, various types of physical activity and sports which particularly appeal to the proprioceptive system (Tai Chi, Yoga, dancing) have been shown to improve balance and to reduce the prevalence of falls in the elderly (Hausdorff et al., 2001; Aydin et al., 2002; Rubenstein and Josephson, 2002; Simmons, 2005). The effect of such activities on balance function is clearly better than that of those aimed to improve cardiovascular function (bioenergetic sports, like running) (Gauchard et al., 2003).

The Korean martial art Taekwondo contains various jumping and weight shifting kicking exercises, which may resemble ballet and gymnastics. Because of these highly dynamic movement patterns, middle-aged or elderly may sustain the largest benefit with regard to dynamic locomotor components, including dynamic balance, from Taekwondo-based training. There are two studies in elderly people on the effects of Taekwondo training on a number of physical fitness components, including balance. Brudnak et al. trained six senior subjects (63–81 year) once a week during 17 weeks. They found an average increase of 16 s in the single leg stance test, which they used as a measure of balance. The authors concluded that Taekwondo training is possible in senior people, and that the profit they found would probably increase and last longer with longer training periods (Brudnak et al., 2002). Cromwell et al. investigated 20 elderly (mean age 72.5 year) who followed 1-h Taekwondo classes two times a week during 11 weeks. A number of parameters related to balance and walking pattern improved significantly in the Taekwondo group, but not in controls of the same age (Cromwell et al., 2007). Although the results are interesting, program duration in these two studies was short, the number of subjects and the number of parameters studied small, and the number of dropouts unclear (Brudnak et al., 2002; Cromwell et al., 2007). Therefore, in a group of healthy, elderly volunteers we studied the effect of 1 year Taekwondo training on several balance parameters, including motor orientation ability, postural balance, and physical tests for static and dynamic balance. If effective such Taekwondo-based exercise program could offer a cheap method to improve balance-dependent locomotor skills of the middle-aged and elderly, which eventually may contribute to the mitigation of the age-related loss of self-maintenance in the general population.

## Materials and Methods

The study was a single arm intervention study in which participants served as their own control.

### Study population

Healthy male and female volunteers, 40–75 years of age. Inclusion criteria: willingness to follow at least a 1-h Taekwondo training session weekly. All volunteers had a routine neurological and cardiologic physical investigation and an exercise ECG. Exclusion criteria: current psychiatric treatment, the use of oral anticoagulants, and/or any disease that was expected to interfere with training, as judged by the volunteer’s treating physician, the study neurologist (J. Lodder), or study cardiologist.

### Recruitment and consent

Posters inviting people for participation were posted at various locations in the Maastricht Hospital and University buildings. Interested people were informed by oral and written information, and they were encouraged to follow at least two Taekwondo sessions at the trainers’ Taekwondo club before enrollment.

### Training sessions

Training intensity was adjusted to the participants’ physical condition. The sessions lasted about 1 h, and generally consisted of the following elements: a short warming-up; Taekwondo techniques, such as stances, blockings, kicks, punches, and strikes; poomsae (style figures), which are fixed patterns, illustrating a fight against one or more imaginary opponents; some elementary self-defense Taekwondo techniques. As Taekwondo combat sparring was considered injury prone, such practice was not included. We practiced the style of the World Taekwondo Federation, and in accord with the guidelines offered by the Kukkiwon, which is the official South Korean governmental Taekwondo governing organization (http://www.wtf.org/). We wrote each week’s training program in advance as a training script, taking account of both the overall aims of the project and the volunteers’ ability to cope with gradually increasing training intensity and complexity. Participants were advised to daily perform some simple balance exercises for 5–10 min, and also to practice some simple stretching exercises. After each training session, we sent an e-mail letter to all participants with a reflection of the latest training session, a preview of the next one, and an invitation to make any comments on any aspect of the program.

The first training session took place on 21 October, 2009, and the final one on 19 January, 2011. We aimed at 40 lessons for each participant within 1 year, but because the attendance rate was lower than anticipated, we decided to extent the training period with 3 months. During the study, several participants chose to follow additional Taekwondo lessons at our Taekwondo club facility, where we adapted the club training program to comply with the study protocol. Number of lessons at the two locations was recorded separately.

### Withdrawal

Subjects could leave the study at any time without any obligation to reveal their reasons, but we listed such reasons to evaluate the feasibility of the training program. We encouraged study participation by creating an informal atmosphere, and we reserved ample time for feedback.

### Measurements

All participants were tested in the month before the start of the study, and as soon as possible after the last training session.

*A questionnaire* on the participants’ subjective assessment on various aspects of the program was filled out 3 months after study onset, and at its end.

*Single leg stance test* served to measure static balance (Rikli and Edwards, 1991; Hong et al., 2000). While wearing shoes, subjects placed their weight bearing foot in a comfortable position on a three times folded 1 cm thick mat of flexible but solid imitation rubber. They were told to keep their eyes open, both hands on their hips, and their non-weight bearing limb in a slightly flexed position, as just not touching the floor. Three test trials were conducted for each leg separately. Duration (in seconds) measurement began when the subject lifted the non-weight bearing foot from the floor, and ended when the foot made contact with the floor.

*One leg hop test* served to measure dynamic balance, with distance in cm (first toe as reference point) as outcome parameter (Augustsson et al., 2004; Ageberg et al., 2008). Subjects wore shoes and could freely move their arms, allowing a more functional execution of the hop (Ageberg et al., 2001). Subjects were instructed to hop as far as possible, while taking off and landing on the same foot. The average of three hops served as final parameter.

*Motor orientation ability* was defined as the subjective proprioceptive horizontal – SPH as follows: subjects stood barefoot and blindfolded on a motor driven platform that was tilted at random over 1°–10° in 2D. Subsequently, by use of a joystick the subject had to adjust the platform orientation back to completely horizontal. The error angle relative to the horizontal was then established in 2D. The output parameters of the SPH was expressed as the mean with standard deviation (SD) of the error angle obtained after 10 trials in both antero-posterior (AP) and medio-lateral (LAT) direction. The relative change of mean AP and mean LAT, and the SD (SDAP and SDLAT) of the error angle were used to express the motor orientation ability (Gauchard et al., 1999, 2003).

*Postural balance* was tested by 40 s computerized measuring of the sway area, sway path, and sway velocity (the latter is only used to calculate the other parameters), with subjects standing barefoot and blindfolded on an instable horizontal platform (stabulometry) with both feet pointing 20° outward, and with both heels about 2 cm apart. Both sway path and sway area were used as balance parameters. The center of pressure (COP) was sampled at 25 Hz, 16 bits. Sway path was defined as the length of the trajectory of the COP divided by the measurement time (mm/s). Sway area was defined as the time integral of the area swept by the COP trajectory with respect to the initial COP, divided by the measurement time (mm^2^/s) (Baratto et al., 2002).

### Static balance

Single limb stance test, which measures the time in seconds one could stand on one leg (Rikli and Edwards, 1991; Hong et al., 2000). There was only one attempt for each leg, while subjects stood barefoot and blindfolded on a horizontal platform.

*Two questionnaire* items relevant to balance:

Since I started practicing Taekwondo training:

- My ability to maintain balance is:
- The “elegance” with which I move is:

(answer options: much worse/worse/unchanged/better/much better).

### Statistics

Sample size estimation was based on the principle outcome parameter, which was the decrease in angle error on the *Motor orientation ability* test, using the following assumptions:

- The mean decrease over the study period would be approximately 0.24°
- The inter-individual SD of the decreases is 0.4° (reflects the extent to which the participants will perform better)
- (One-sided) alpha = 0.05
- Power = 0.8

For such difference to be detected, at least 17 subjects were required.

Data were analyzed using SPSS, version 17.0, SPSS Inc., Chicago, IL, USA. Baseline and final whole group data were compared using Wilcoxon Signed-Rank Test, expressed as average with SD, and paired *T*-test. Data of participants who improved or did worse are expressed as median with range. A *p*-value < 0.05 was considered statistically significant.

## Results

There were 12 men and 12 women; median age was 57 (range: 41–71) years. Table 1 shows individual demographic data. All participants had all baseline and final measurements, so there were no study dropouts. However, three women and two men withdrew early from the training program (non-compliers; participants’ nr. 1, 14, 20, 22, 23). In two the reason was training related, in two due to external reasons, and in the fifth a combination of both. The demographic characteristics of the non-compliers were similar to those of the subjects completing the study.

**Table 1:** **Participants demographics**.

Nr	Gender	Age	Education	Physical
			level	Activity (h/week)
1	m	61	U	15
2	m	61	U	10
3	m	62	U	0
4	m	59	U	7
5	f	41	U	0
6	m	58	U	9
7	m	51	U	4
8	f	49	H	3
9	f	60	U	10
10	m	66	O	10
11	f	50	H	5
12	m	57	U	4
13	f	51	H	3
14	f	63	U	0
15	m	70	U	2
16	m	53	U	5
17	f	50	H	5
18	m	52	H	3
19	f	57	H	4
20	m	59	U	0
21	f	60	U	1
22	f	44	H	10
23	f	51	H	1
24	f	48	U	0

*U = master degree or equivalent, H = bachelor degree or equivalent, O = other, Gray = non-complier*.

The one leg hop and single leg stance test were conducted in 17 participants. One participant missed the baseline test because he was abroad at that time. The other missed the final test due to a non-training related shoulder injury. The other balance measurements were conducted in all 19 study compliers.

Results for baseline and final measures for all the participants are shown in Table 2.

**Table 2:** **Outcome measurements in study compliers**.

Measurement	Pre mean	SD	Post mean	SD	Significance
Single leg stance (sec)					
Right leg	34.23	19.6	50.35	14.5	0.005
Left leg	35.26	19.4	49.28	16.3	0.007
One leg hop (cm)	100.74	27.4	110.23	39.61	0.280
Motor orientation ability (°)					
Anterio-posterior	0.09	0.9	−0.52	0.9	0.022
SD anterio-posterior	1.04	0.4	0.93	0.4	0.315
Lateral	0.21	1.0	0.16	0.9	0.837
SD lateral	0.78	0.3	0.60	0.3	0.064
Postural balance					
Swaypath (mm/s)	40.01	11.1	27.80	5.7	0.000
Swayarea (mm^2^/s)	11.66	4.9	8.01	2.5	0.005
Static balance (sec)					
Right leg	5.22	4.6	10.56	6.6	0.004
Left leg	5.53	3.8	9.50	6.0	0.039

*Pre = before Taekwondo training, Post = after Taekwondo training, SD = Standard deviation*.

Examination of balance measures shows significant improvement in several tests. Average time for single leg stance increased with 16.1 s for the right leg and 14.0 s for the left; both differences being statistically significant. Considering the right leg, 15 participants improved with a median of 22.8 s (range 5.3–52.0), and two deteriorated with 17.5 s (range 5.5–29.4), whereas there was a median improvement of 24 s in 15 participants (range 0.5–46), and a deterioration of 16.8 s (range 9.8–23.8) in 2 participants for the left leg stance.

The average one leg hop test distance increased (not statistically significant) with 9.5 cm. Eleven participants had a median increased distance of 13.7 cm (range 1–70), whereas in six there was a median decrease of 18.7 cm (range 0.7–43.5).

Motor orientation ability significantly increased in favor of the AP direction with a difference of 0.62° toward anterior compared to pre-training measurement, when the participants corrected the tilted platform rather toward the posterior direction. The error in adjusting the platform back to the horizontal (SD) did not change significantly neither in the anterior-posterior nor in the lateral direction. In total 15 participants improved with a median decrease of 0.7° (range 2.55–0.14) and 4 participants did worse with a median increase of 0.53° (range 0.36–0.99) in AP direction. In the lateral direction 10 participants improved with a median decrease of 0.7° (range 2.97–0.7) and 9 participants did worse with an increase of 0.7° (range 0.01–1.68).

On postural balance measurements, both sway path and sway area improved significantly as shown in Table 1. Sway path improved in all 19 participants, with a median of 9.3 mm/s (range 0.71–45.86). Sway area improved in 15 participants with 4.2 mm^2^/s (range 17.39–1.22) and increased in 4 participants with 1.7 mm^2^/s (range 0.79–3.97).

Static balance improved with an average duration of standing on the right leg with 5.34 s, and with almost 4 s for the left leg, which were statistical significantly longer durations compared with pre-training measurements. Median right leg standing duration increased in 17 participants with 5 s (range 1–16), whereas it decreased in 2 participants with 6.5 s (range 1–12). Median left leg standing duration increased in 13 participants with 8 s (range 1–18), whereas it decreased in 6 participants with 3.5 s (range 2–6).

The questionnaire reported a *better* “ability to maintain balance” in 16 of the 19 compliers, whereas it was *unchanged* in three. Three of the 19 study compliers reported *better* “elegance” of their movements, whereas 16 reported this item was *unchanged*. All non-compliers reported unchanged on both items.

To explore, whether age, gender, or the degree of non-study physical activity independently affected the differences we found, we used linear regression modeling with the primary outcome measure the Anterior-Posterior direction error angle of the Motor Orientation Ability Test as dependent variable, and as independent variables age, gender, and hours of non-study physical activity. Only gender appeared as independently affecting outcome (*p* = 0.043). When baseline AP measurements were added to the model, the baseline AP instead of gender appeared independently (*p* = 0.03) related to the increased AP forward correction. Exploring single leg stance outcome data yielded non-test physical activity independently inversely related for only the left leg (*p* = 0.034), and also only for the left leg in the static balance test (*p* = 0.050). Exploring sway path and sway area did not yield any of the independent variables to be associated with test outcome.

## Discussion

Our data show that a 1-year weekly Taekwondo training improves various aspects of balance in middle-aged healthy people. Motor orientation ability was the primary endpoint measure we based our sample size estimation on, but the assumed improvement of a decrease in the angle in the direction of correction was not achieved. However, much to our surprise we observed a significant forward directed correction independent of the original direction of platform correction. None of the five non-compliers demonstrate a change in preferred direction of platform correction. Our data, therefore, suggest that Taekwondo training improves balance control by increased correction toward forward direction. Not only is leaning forward in an upright position more stable than leaning backwards, but our result concurs with the “optimal bending” model presented in Alexandrov et al. in which forward bending is taken as an anticipatory postural adjustment (Massion, 1992; Alexandrov et al., 2001).

Increased loss of balance in the elderly may relate to an age-related increase of posterior sway at the expense of a decrease of forward sway which is much more effective in balance maintenance. Younger people use the forefoot’s muscle activity for maintenance of balance more intensely than elderly, who rather tend to use heel area function in the posterior sway. As the maximal range of forward sway to maintain balance is twice as large as in backward sway (8° versus 4°), elderly are more prone to fall, and when they do it is often backwards (Tanaka et al., 1999). This shift toward more intensive use of forward control in balance control after Taekwondo training likely results from the typical stances, movement in such stances, and the footwork in Taekwondo exercises, as these movements require the center of gravity to be balanced by mainly forefoot function (Leong et al., 2011).

From our findings we may hypothesize that age-adapted Taekwondo training may contribute to lowering the chance of falls in the elderly. Even if in case of an imposed disequilibrium trained subjects may not be able to correct this completely, they may fall forward rather than backward, which may be less hazardous than falling backwards (Choi et al., 2011).

Although our study was not powered to any *a priori* hypothesis on a differential effect between men and women, *post hoc* exploration by means of linear regression analysis suggested that especially women may benefit from the training, as female gender emerged as independently related to the outcome measure. This finding may relate to a lower initial forward correction in females than in males. In fact, the average initial correction was 0.62° backwards in women, which improved to 0.35 forwards (difference: 0.97), whereas men increased their initial average forward correction of 0.37–0.68 (difference: 0.31). One may speculate about the cause of these differences, but we should realize that these are *post hoc* findings in a small group. In any case our findings stress the importance of accounting for the possibility of substantial differences between balance performances based on gender, and future studies should be sufficiently powered to account for such difference.

Sway path and sway area both decreased significantly, which was probably related to the fact the center of gravity had moved forwards, with a higher plantar pressure providing stronger sensory stimulation to the mechanoreceptors. This effect would result in an overall increased neural feedback from the cutaneous receptors to the central nervous system, and possibly contribute to improved postural control (Qiu et al., 2012). Subjective judgment by our volunteers on balance control was in line with measurement data. Linear regression did not detect any independently related outcome predictors for sway path and sway area.

Another way we measured balance and which had improved after training was the single leg stance, both with eyes open and closed. Dependence upon visual information for balance maintenance increases with age (Gauchard et al., 2001). Our data suggest that Taekwondo training improves postural control by improving vestibular or proprioceptive input, or both, as participants were less dependent on visual information at the end of the study. These data may implicate that during walking single leg support is prolonged and the duration of double leg support shortened. Such dynamic locomotor components decrease with age: steps become shorter, walking pace decreases, the time that both feet touch the ground during walking increases (double support) (Gabell et al., 1985; Tinetti et al., 1988; Hornbrook et al., 1994; Hausdorff et al., 2001; Rubenstein and Josephson, 2002). Therefore, Taekwondo training may improve stable walking with a decrease in the chance of falling accidents when people get older. However, this remains hypothetical as we did record neither the duration of single or double leg support during walking nor the frequency of falling accidents in our volunteers. The only factor that came out as independently associated in both leg standing performance tests, but only for the left leg, was the number of hours of non-study physical activity, in this sense that a lower physical activity was associated with more improvement. This finding may suggest that the non-dominant leg may become more involved in static balance maintenance by Taekwondo training in physically non-active people but that such improvement may not be specific for Taekwondo.

The one leg hop test showed a (statistically non-significant) mean distance increase of 9.02 cm. It should be reminded that this test was developed for measurements in patients with anterior cruciate ligament reconstruction (Tegner et al., 1986). Because apart from joint stability it encompasses clinical attributes such as neuromuscular coordination and proprioception we used it to test (dynamic) balance. However, the test has not been validated for balance, and may not serve as a dynamic balance measure.

The effects of the training program on balance control could not be related to an increase of leg muscle power as measured with a Biodex System 3 Pro dynamometer. We found no increase in either isometric or isokinetic measurement data of quadriceps or hamstring muscles, which suggests that changes rather took place at neural or vestibular level.

Beneficial effects of training on balance control may not be specific for Taekwondo, as other martial arts or even mere physical activity may have such effects (Perrin et al., 1999). One of the martial arts that has been studied frequently and had positive effects on balance is Tai Chi (Maciaszek and Osinski, 2010), although the desired practical implications such as a lower risk of fall accidents do not simply seem to follow from such effects (Logghe et al., 2009). However, any direct comparison of studies remains difficult because of differences in various aspects of study design and outcome parameters. Taekwondo differs especially from Tai Chi regarding movement dynamics, and eventually elderly who are able to perform more dynamic movement patterns may opt for age-adjusted Taekwondo or otherwise choose for Tai Chi.

Our study suffers some limitations. First, it was not randomized. Although theoretically unknown factors might have contributed to the improvements that we found, we consider this unlikely as we would not expect parameters to improve with a year of further aging. Therefore, we think it unlikely to have measured random noise. Second, considering the generally high education level of our participants, and the fact that most of them were already physically active in other ways, some caution is warranted when extrapolating our results to middle-aged people in general (Pons van Dijk et al., accepted). Third, there were five non-compliers, but we were still able to conclude the study with more participants than the sample size estimation required.

In conclusion, our data suggest that age-adapted Taekwondo training improves various aspects of balance control in healthy people over the age of 40. Recently we reported on the feasibility and safety of such training program (Pons van Dijk et al., accepted). Whether long-term adherence to age-adapted Taekwondo training indeed has a beneficial effect on mobility and fall accident frequency in people with a higher age remains for further study.

## Conflict of Interest Statement

The authors declare that the research was conducted in the absence of any commercial or financial relationships that could be construed as a potential conflict of interest.

## References

[B1] Ageberg E. Thomeé R. Neeter C. Silbernagel K. G. Roos E. M. (2008). Muscle strength and functional performance in patients with anterior cruciate ligament injury treated with training and surgical reconstruction or training only: a two to five-year followup. Arthritis Rheum. 59, 1773–1779 10.1002/art.24066 19035430

[B2] Ageberg E. Zätterström R. Fridén T. Moritz U. (2001). Individual factors affecting stabilometry and one-leg hop test in 75 healthy subjects, aged 15-44 years. Scand. J. Med. Sci. Sports 11, 47–53 10.1034/j.1600-0838.2001.011001047.x 11169235

[B3] Alexandrov A. Frolov A. A. Massion J. (2001). Biomechanical analysis of movement strategies in human forward trunk bending. II. Experimental study. Biol. Cybern. 84, 435–443 10.1007/PL00007986 11417055

[B4] Augustsson J. Thomeé R. Karlsson J. (2004). Ability of a new hop test to determine functional deficits after anterior cruciate ligament reconstruction. Knee Surg. Sports Traumatol. Arthrosc. 12, 350–356 10.1007/s00167-004-0518-4 15138668

[B5] Aydin T. Yildiz Y. Yildiz C. Atesalp S. Kalyon T. A. (2002). Proprioception of the ankle: a comparison between female teenaged gymnasts and controls. Foot Ankle Int. 23, 123–129 11858332 10.1177/107110070202300208

[B6] Baratto L. Morasso P. G. Re C. Spada G. (2002). A new look at posturographic analysis in the clinical context: sway-density versus other parameterization techniques. Motor Control 6, 246–270 12122219 10.1123/mcj.6.3.246

[B7] Brudnak M. A. Dundero D. Van Hecke F. M. (2002). Are the “hard” martial arts, such as the Korean martial art, TaeKwon-Do, of benefit to senior citizens? Med. Hypotheses 59, 485–491 10.1016/S0306-9877(02)00203-7 12208194

[B8] Choi C. J. Lim H. W. Park M. K. Cho J. G. Im G. J. Chae S. W. (2011). Does the kyphotic change decrease the risk of fall? Clin. Exp. Otorhinolaryngol. 4, 118–121 10.3342/ceo.2011.4.3.126 21949576 PMC3173701

[B9] Cromwell R. L. Meyers P. M. Meyers P. E. Newton R. A. (2007). Taekwondo: an effective exercise for improving balance and walking ability in older adults. J. Gerontol. A Biol. Sci. Med. Sci. 62, 641–646 10.1093/gerona/62.6.641 17595421

[B10] Gabell A. Simons M. A. Nayak U. S. (1985). Falls in the healthy elderly: predisposing causes. Ergonomics 28, 965–975 10.1080/00140138508963219 4043031

[B11] Gauchard G. C. Gangloff P. Jeandel C. Perrin P. P. (2003). Influence of regular proprioceptive and bioenergetic physical activities on balance control in elderly women. J. Gerontol. A Biol. Sci. Med. Sci. 58, 846–850 10.1093/gerona/58.9.M846 14528042

[B12] Gauchard G. C. Jeandel C. Perrin P. P. (2001). Physical and sporting activities improve vestibular afferent usage and balance in elderly human subjects. Gerontology 47, 263–270 10.1159/000052810 11490145

[B13] Gauchard G. C. Jeandel C. Tessier A. Perrin P. P. (1999). Beneficial effect of proprioceptive physical activities on balance control in elderly human subjects. Neurosci. Lett. 273, 81–84 10.1016/S0304-3940(99)00615-1 10505621

[B14] Hausdorff J. M. Rios D. A. Edelber H. K. (2001). Gait variability and fall risk in community-living older adults: a 1-year prospective study. Arch. Phys. Med. Rehabil. 82, 1050–1056 10.1053/apmr.2001.24893 11494184

[B15] Hong Y. Li X. J. Robinson P. D. (2000). Balance control, flexibilty, and cardiorespiratory fitness among older tai chi practitioners. Br. J. Sports Med. 34, 24–29 10.1136/bjsm.34.1.29 PMC1724150 10690447

[B16] Hornbrook M. C. Stevens V. J. Wingfield D. J. Hollis J. F. Greenlick M. R. Ory M. G. (1994). Preventing falls among community-dwelling older persons: results from a randomized trial. Gerontologist 34, 16–23 10.1093/geront/34.1.16 8150304

[B17] Ishiyama G. (2009). Imbalance and vertigo: the aging human vestibular periphery. Semin. Neurol. 29, 491–499 10.1055/s-0029-1241039 19834860

[B18] Leong H. T. Fu S. N. Ng G. Y. Tsang W. W. (2011). Low-level Taekwondo practitioners have better somatosensory organisation in standing balance than sedentary people. Eur. J. Appl. Physiol. 111, 1787–1793 10.1007/s00421-010-1798-7 21221991

[B19] Logghe I. H. Zeeuwe E. Verhagen A. P. Wijnen-Sponselee R. M. Willemsen S. P. Bierma-Zeinstra S. M. (2009). Lack of effect of Tai Chi Chuan in preventing falls in elderly people living at home: a randomized clinical trial. J. Am. Geriatr. Soc. 57, 70–75 10.1111/j.1532-5415.2008.02064.x 19054193

[B20] Maciaszek J. Osinski W. (2010). The effects of Tai Chi on body balance in elderly people – a review of studies from the early 21st century. Am. J. Chin. Med. 38, 219–229 10.1142/S0192415X10007798 20387220

[B21] Massion J. (1992). Movement, posture and equilibrium: interaction and coordination. Prog. Neurobiol. 38, 35–65 10.1016/0301-0082(92)90034-C 1736324

[B22] Nasher L. (1997). “Practical biomechanics and physiology of balance,” in Handbook of Balance Function Testing, eds Jacobson G. Newman C. W. Kartush J. M. (San Diego: Singular Publishing Group), 261–279

[B23] Perrin P. P. Gauchard G. C. Perrot C. Jeandel C. (1999). Effects of physical and sporting activities on balance control in elderly people. Br. J. Sports Med. 33, 21–126 10.1136/bjsm.33.2.121 PMC1756147 10205695

[B24] Qiu F. Cole M. H. Davids K. W. Hennig E. M. Silburn P. A. Netscher H. (2012). Enhanced somatosensory information decreases postural sway in older people. Gait Posture 35, 630–635 10.1016/j.gaitpost.2011.12.013 22245163

[B25] Rikli R. E. Edwards D. J. (1991). Effects of a three-year exercise program on motor function and cognitive processing speed in older women. Res. Q. Exerc. Sport 62, 61–67 10.1080/02701367.1991.10607519 2028094

[B26] Rubenstein L. Z. Josephson K. (2002). The epidemiology of falls and syncope. Clin. Geriatr. Med. 18, 141–158 10.1016/S0749-0690(02)00002-2 12180240

[B27] Simmons R. (2005). Sensory organization determinants of postural stability in trained ballet dancers. Int. J. Neurosci. 115, 87–97 10.1080/00207450490512678 15768854

[B28] Tanaka T. Takeda H. Izumi T. Ino S. Ifukube T. (1999). Effects on the location of the centre of gravity and the foot pressure contribution to standing balance associated with ageing. Ergonomics 42, 997–1010 10.1080/001401399185261 10424187

[B29] Tegner Y. Lysholm J. Lysholm M. Gillquist J. (1986). A performance test to monitor rehabilitation and evaluate anterior cruciate ligament injuries. Am. J. Sports Med. 14, 156–159 10.1177/036354658601400212 3717488

[B30] Tinetti M. E. Speechley M. Ginter S. F. (1988). Risk factors for falls among elderly persons living in the community. N. Engl. J. Med. 319, 1701–1707 10.1056/NEJM198812293192604 3205267

[B31] Wolter L. L. Studenski S. A. (1996). A clinical synthesis of falls intervention trials. Top. Geriatr. Rehabil. 11, 9–19

